# Antipsychotic Medications and Risk of Acute Coronary Syndrome in Schizophrenia: A Nested Case-Control Study

**DOI:** 10.1371/journal.pone.0163533

**Published:** 2016-09-22

**Authors:** Hsing-Cheng Liu, Shu-Yu Yang, Ya-Tang Liao, Chiao-Chicy Chen, Chian-Jue Kuo

**Affiliations:** 1 Department of General Psychiatry, Taipei City Psychiatric Center, Taipei City Hospital, Taipei, Taiwan; 2 Department of Psychiatry, School of Medicine, Taipei Medical University and Psychiatric Research Center, Taipei Medical University Hospital, Taipei, Taiwan; 3 Department of Pharmacy, Taipei City Psychiatric Center, Taipei City Hospital, Taipei, Taiwan; 4 Graduate Institute of Clinical Pharmacy, College of Pharmacy, Kaohsiung Medical University, Kaohsiung, Taiwan; 5 Institute of Epidemiology and Preventive Medicine, College of Public Health, National Taiwan University, Taipei, Taiwan; 6 Department of Psychiatry, Mackay Memorial Hospital, Taipei, Taiwan; University of Manitoba, CANADA

## Abstract

**Background:**

This study assessed the risk of developing acute coronary syndrome requiring hospitalization in association with the use of certain antipsychotic medications in schizophrenia patients.

**Methods:**

A nationwide cohort of 31,177 inpatients with schizophrenia between the ages of 18 and 65 years whose records were enrolled in the National Health Insurance Research Database in Taiwan from 2000 to 2008 and were studied after encrypting the identifications. Cases (n = 147) were patients with subsequent acute coronary syndrome requiring hospitalization after their first psychiatric admission. Based on a nested case-control design, each case was matched with 20 controls for age, sex and the year of first psychiatric admission using risk-set sampling. The effects of antipsychotic agents on the development of acute coronary syndrome were assessed using multiple conditional logistic regression and sensitivity analyses to confirm any association.

**Results:**

We found that current use of aripiprazole (adjusted risk ratio [RR] = 3.68, 95% CI: 1.27–10.64, p<0.05) and chlorpromazine (adjusted RR = 2.96, 95% CI: 1.40–6.24, p<0.001) were associated with a dose-dependent increase in the risk of developing acute coronary syndrome. Although haloperidol was associated with an increased risk (adjusted RR = 2.03, 95% CI: 1.20–3.44, p<0.01), there was no clear dose-dependent relationship. These three antipsychotic agents were also associated with an increased risk in the first 30 days of use, and the risk decreased as the duration of therapy increased. Sensitivity analyses using propensity score-adjusted modeling showed that the results were similar to those of multiple regression analysis.

**Conclusions:**

Patients with schizophrenia who received aripiprazole, chlorpromazine, or haloperidol could have a potentially elevated risk of developing acute coronary syndrome, particularly at the start of therapy.

## Introduction

Schizophrenia is the most deliberating mental illness and has a tremendous impact on a person’s psychosocial functioning. In addition, schizophrenia patients are more prone to several medical comorbidities than the general population, especially metabolic syndrome, pneumonia, and cardiovascular disorders [1–3]. Medical comorbidities could be attributable to several factors, such as smoking, high alcohol consumption, malnutrition, poor personal hygiene, lack of exercise, and the antipsychotic drugs taken [1, 4, 5]. Compared with the general population, patients with schizophrenia had a two-fold higher prevalence of cardiovascular disorders [6] which could result in higher risk for cardiovascular related mortality [7].

Coronary heart diseases derived from the occlusion of coronary arteries comprise more than half of all cardiovascular events [8]. Acute coronary syndrome is an acute, severe, life-threatening ischemic event and includes unstable angina and acute myocardial infarction [9–11].

Metabolic syndrome induced by second-generation antipsychotic drugs in patients with schizophrenia is well characterized in prior studies [12, 13]. Clozapine is associated with the highest risk of developing metabolic syndrome, while olanzapine and risperidone carry intermediate risk of it [13]. Metabolic syndrome is one of the crucial risk factors predisposing patients to cardiovascular diseases [12]. Therefore, we suggest second-generation antipsychotics with higher risks of metabolic syndrome might also carry a greater risk of acute coronary syndrome. A recent study [14] reported that antipsychotic used in the elderly population is associated with a decreased risk of hospitalization for acute coronary syndrome. As for myocardial infarction, one review article [1] suggested the association between antipsychotics and risk of acute coronary syndrome is inconsistent. Several studies [15–18] reported that first-generation antipsychotic agents have a moderate to strong effect on the risk of myocardial infarction. In contrast, a large-scale study [19] reported no association between the risk of myocardial infarction and current use of either first- or second-generation antipsychotic agents. Reasons for the inconsistencies include the heterogeneity of antipsychotic agents, differences in patient populations, and potential uncontrolled confounders (such as inherent physical illnesses). Thus, further studies are needed to examine the associations.

We conducted a nested case-control study of a nationwide schizophrenia cohort in Taiwan to estimate the associations of antipsychotic agents and risk of acute coronary syndrome. We explored the risk in association with individual antipsychotics and used propensity-scoring methods as sensitivity analyses to confirm the associations.

## Materials and Methods

### Study subjects

The data source is described in detail elsewhere [20–22]. Therefore, we briefly summarize it here. Taiwan initiated a single-payer National Health Insurance program on March 1, 1995, and nearly 98% of the entire Taiwanese population (23 million) is enrolled [20]. In 1996, the National Health Research Institute in Taiwan established the National Health Insurance Research Database to provide data for research purposes. Please find the website (http://nhird.nhri.org.tw/en/) to request the information this study used, such as a point of contact and data files. The database constitutes medical claim files representative of the entire population in Taiwan [20].

We used the Psychiatric Inpatient Medical Claims database, which is a subset of the National Health Insurance Research Database. The Psychiatric Inpatient Medical Claims database consists of all patients hospitalized for any psychiatric illness from 1996 through 2008 (N = 187,117). The database includes patients with one or more inpatient psychiatric hospitalization records and, at least, one discharge diagnosis of a mental illness based on the International Classification of Diseases, Ninth Revision (ICD-9) codes 290 to 319. The database contains patients’ demographics, diagnoses, prescription claims data, and medical expenditures [20–22].

The patients eligible for enrollment into the study had, at least, one hospitalization for a psychiatric illness between 2000 and 2008, but no psychiatric hospitalizations from 1996 through 1999 (N = 125,225)[23] (Fig A in S1 File). The inclusion criterion was that each patient’s diagnosis at discharge fulfilled the principal diagnosis of schizophrenia (ICD-9 code 295.xx). Additionally, patients must have been 18 to 65 years of age at their first psychiatric hospital admission. We excluded patients with the diagnoses of ischemic heart disease, myocardial infarction, and coronary artery disease (International Classification of Diseases [ICD] 9 codes 410–414) before their first psychiatric admission. All of the medical claims data from 1996 and 2010 were retrieved from included patients after encryption to protect patient identity.

### Ethics statement

Patient identification was protected by the confidentiality policy of the Bureau of National Health Insurance, Taiwan [21], and database users each signed a contract guaranteeing patient confidentiality before access was granted. A waiver of informed consent was granted due to the minimal risk to the privacy of individual subjects since the patient records/information was anonymized and de-identified before patient selection and analysis. The Institutional Review Board of the Committee on Human Subject Research of Taipei City Hospital reviewed and approved this study.

### Case and control definitions

Patients with subsequent acute coronary syndrome requiring hospitalization after their first psychiatric admission were identified as cases (N = 148). Acute coronary syndrome comprised acute myocardial infarction (410.xx) (N = 101) and intermediate coronary syndrome (411.1x) (N = 47), for which the definition is widely applied [9–11] and strict [24, 25]. A nested case-control design was employed, and cases and controls were selected from the cohort. Based on risk-set sampling, 20 controls for each case were randomly selected from the cohort and matched by sex, age (±5 years), and the year of the first psychiatric admission. The index date for each case was defined as the date of hospitalization for acute coronary syndrome.

We assigned the same index date to the controls as their matching case, which was later than the first psychiatric admission of each control. Additionally, each control had, at least, one claim record after the corresponding index date to confirm that patients remained alive at the date. Subsequently, 147 case-control sets (i.e., 147 cases and 2940 controls) were included. One case was eliminated because an adequate number of controls could not be found.

### Exposure definition

The antipsychotic drug use data was compiled from the prescription files of all outpatient and emergency visits and hospitalizations. The duration and dosage of each antipsychotic drug were calculated based on each prescription record, which contains the type of medication, dosage, time of prescription, and duration of drug supply. Drug exposure in each case-control pair in association with the risk of acute coronary syndrome was evaluated by several approaches. First, current users were defined as the patients taking a specified drug during the 30 days before the index date [20]; the remainder was defined as noncurrent users and served as the reference group in the analysis.

In this study, only the antipsychotic drugs with substantial use by current users (> 3%) were included in the individual drug analyses. Therefore, the second-generation antipsychotics analyzed included clozapine, olanzapine, quetiapine, zotepine, risperidone, amisulpride, and aripiprazole. The commonly used first-generation antipsychotics included chlorpromazine, haloperidol, flupentixol, and sulpiride. Due to the longer-acting effects of standard injectable neuroleptics compared with the corresponding oral preparations, we extended the current use period to account for these long-acting antipsychotic agents. For example, the standard injection intervals of haloperidol decanoate and long-acting injectable risperidone were 28 and 14 days, respectively [26]. Therefore, the current use periods for haloperidol decanoate and long-acting injectable risperidone were 58 and 44 days, respectively, instead of 30 days. For short-acting injectable neuroleptic agents, the effective periods were the same as their oral preparations. We then estimated the temporal relationship of the association between the specified drug exposure and acute coronary syndrome. Second, for investigating the dose-dependent relationship, we estimated the relationship using two dimensions of drug receipt data, i.e., the duration of the drug used in the current period, and the cumulative defined daily dose in the current period. The defined daily dose (DDD) was based on the dose information obtained from the Anatomical Therapeutic Chemical Classification System (ATC/DDD Index 2015. http://www.whocc.no/atc_ddd_index/ [accessed March 1, 2015]) [27]. For example, 15 mg of aripiprazole was equivalent to one DDD. Third, we estimated the effect of continuous antipsychotic exposure on the risk of acute coronary syndrome. The method for calculation of continuous antipsychotic exposure was used in a prior study [20], and we describe it briefly here. In current users, the numbers of days of antipsychotic drug exposure starting with the prescribing date closest to the index date were retrospectively added together as the total continuous exposure days; 30 days between two prescriptions constituted a treatment gap, and the previous exposure was not added to the total.

### Potential confounders

The matching process of the study design inherently controlled for age and gender. We adjusted for the Charlson comorbidity score at the first psychiatric admission. The Charlson comorbidity index is the sum of the weighted scores of 31 comorbid conditions used for assessing general health status [28]. Concurrent medications prescribed and comorbid physical illnesses appearing within 6 months before the index date [20–22] (Table 1) were also adjusted.

**Table 1:** Characteristics of case patients with acute coronary syndrome and control patients derived from a nationwide cohort with schizophrenia (N = 31,177).

Characteristic, n (%)	Cases (n = 147)	Controls (n = 2940)	P-value^a^
**At first admission**	Mean (SD)	Mean (SD)	
Age, years	45.7 (12.2)	45.6 (12.0)	0.131
	n (%)	n (%)	
Men	94 (64.0)	1880 (64.0)	–
Charlson comorbidity index			
1	102 (69.4)	2457 (83.6)	Reference
2	35 (23.8)	400 (13.6)	<0.001
≥3	10 (6.8)	83 (2.8)	0.002
**Within 180 days before the index date**			
Physical illnesses			
Diabetes mellitus	48 (32.7)	276 (9.4)	<0.001
Cerebrovascular disease	18 (12.2)	51 (1.7)	<0.001
Chronic hepatic disease	16 (10.9)	155 (5.3)	0.004
Hypertension	57 (38.8)	354 (12.0)	<0.001
Asthma	10 (6.8)	48 (1.6)	<0.001
Concomitant drugs			
Drugs used in diabetes	48 (32.7)	249 (8.5)	<0.001
Antihypertensives	19 (12.9)	69 (2.4)	<0.001
Agents acting on the renin-angiotensin system	59 (40.1)	185 (6.3)	<0.001
Lipid modifying agents	36 (24.5)	125 (4.3)	<0.001
Corticosteroids for systemic use	39 (26.5)	260 (8.8)	<0.001
Nasal preparations	31 (21.1)	449 (15.3)	0.057
Drugs for obstructive airway diseases	54 (36.7)	460 (15.7)	<0.001
Cough and cold preparations	83 (56.5)	1099 (37.4)	<0.001
Antihistamines for systemic use	71 (48.3)	972 (33.1)	<0.001
Mood stabilizer	26 (17.7)	467 (15.9)	0.560
Antidepressant	37 (25.2)	571 (19.4)	0.088

^a^Estimated using univariate conditional logistic regression.

### Statistical analysis

The crude incidence of acute coronary syndrome in the cohort was calculated as the number of incident cases divided by the sum of each subject’s contributed person-years. The contributed person-years for each subject were calculated from the date of a patient’s first psychiatric admission to the date of incident acute coronary syndrome, death, or December 31, 2010. Differences in the incidences of acute coronary syndrome between males and females were investigated using life table survival analysis. Univariate conditional logistic regression was used for the comparisons between cases and controls initially. Then, multivariate regression was employed to estimate the effects of individual antipsychotic drugs on the risk of acute coronary syndrome. Regression analyses were conducted using SAS software, version 9.2 (SAS Institutes Inc., Cary, NC, USA). A p value of 0.05 was considered significant.

### Sensitivity analysis

The assignment of antipsychotic drugs was determined by the attending psychiatrist depending on the presence of comorbid physical illnesses and concomitant medications (Table 1). Therefore, we ran propensity score-adjusted regressions [29] to determine the potential influence of the covariates on the estimates of the associations involving the individual antipsychotic drugs and acute coronary syndrome. In addition, we conducted subgroup analysis by restricting the outcome to acute myocardial infarction (ICD 9 codes 410.xx), which is the most severe form of acute coronary syndrome, to confirm any association. Furthermore, for handling multiple analyses conservatively, we conducted a sensitivity analysis with the p value set at 0.01 for significance and then interpreted the results.

## Results

The study cohort included 31,177 patients with schizophrenia that met the enrollment criteria. The incidence of acute coronary syndrome requiring hospitalization was 6.93 cases per 10,000 person-years in patients with schizophrenia (95% CI = 5.86–8.14, based on the Poisson distribution). Men had a higher incidence of acute coronary syndrome than women did but it was not statistically significant [8.07 (95% CI = 6.53–9.87) vs. 5.67 (95% CI = 4.25–7.42) per 10,000 person-years].

### Comorbid physical illnesses and concomitant medications

Table 1 presents the clinical attributes of the case patients with acute coronary syndrome and the control patients. Cases had higher Charlson comorbidity scores at the first psychiatric admission than controls. In addition, cases had greater numbers of physical illnesses and concomitant medications 180 days before the index date than controls did.

### Temporal relationship

**Fig 1:**
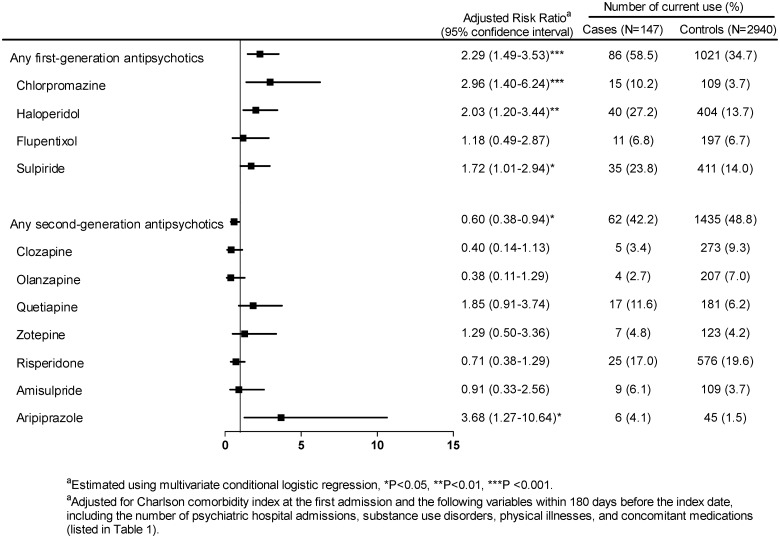
Association between the current use of each second- or first-generation antipsychotic agent and the risk of acute coronary syndrome relative to the noncurrent use of antipsychotic agent (reference group).

After adjustment for the variables in Table 1 (except for gender and age), Fig 1 shows that relative to noncurrent use, current use of any second-generation antipsychotic agent was inversely associated with the risk of acute coronary syndrome. In contrast, use of any first-generation antipsychotic agent was associated with an increased risk of acute coronary syndrome (adjusted risk ratio [RR] = 2.29, 95% CI: 1.49–3.53, p < 0.001).

Of the second-generation antipsychotics analyzed, aripiprazole was the only one with a statistically significant risk of acute coronary syndrome (adjusted RR = 3.68, 95% CI: 1.27–10.64, p < 0.05). Of the first-generation antipsychotics analyzed, chlorpromazine carried a higher risk of acute coronary syndrome, followed by haloperidol and then sulpiride. Other individual antipsychotics were not associated with the risk of acute coronary syndrome.

### Dose-dependent relationship

Table 2 shows that the duration for current use of aripiprazole or chlorpromazine was significantly associated with the risk of acute coronary syndrome. Furthermore, on a defined daily dose basis among current antipsychotic users, aripiprazole was the only antipsychotic associated with the risk of the syndrome.

**Table 2:** Duration of therapy and dose of individual antipsychotic agents by case patients with acute coronary syndrome and controls.

		Cases (n = 147)		Controls (n = 2940)	Multivariate Adjusted Model		
Characteristic	n (use)	Mean (SD) of all cases	N (use)	Mean (SD) of all controls	Adjustedrisk ratio^a^	95% CI	P-value
Current users (duration of use within 30 days before the index date, days)							
Chlorpromazine	15	2.4 (7.6)	109	0.7 (4.0)	1.05**	1.02–1.09	0.002
Haloperidol	40	3.5 (7.8)	404	2.1 (6.7)	1.01	0.98–1.04	0.388
Flupentixol	11	0.6 (4.0)	197	0.7 (4.2)	1.03	0.98–1.08	0.298
Sulpiride	35	3.8 (8.2)	411	2.6 (7.6)	1.02	0.99–1.05	0.135
Clozapine	5	0.5 (3.1)	273	1.5 (5.9)	0.96	0.91–1.02	0.200
Olanzapine	4	0.3 (1.8)	207	1.4 (5.7)	0.91	0.80–1.04	0.165
Quetiapine	17	1.8 (5.4)	181	1.2 (5.2)	1.02	0.99–1.06	0.212
Zotepine	7	0.9 (4.3)	123	0.7 (3.9)	1.02	0.98–1.07	0.325
Risperidone	25	2.1 (6.2)	576	3.2 (8.1)	0.97	0.93–1.01	0.127
Amisulpride	9	1.1 (5.1)	109	0.7 (4.0)	0.99	0.94–1.04	0.786
Aripiprazole	6	0.8 (4.3)	45	0.3 (2.7)	1.06*	1.01–1.11	0.013
Current users (cumulative defined daily dose within 30 days before index date, DDD)							
Chlorpromazine	15	0.7 (3.0)	109	0.3 (2.9)	1.04	1.00–1.08	0.079
Haloperidol	40	2.6 (11.4)	404	2.4 (11.0)	1.00	0.99–1.02	0.667
Flupentixol	11	0.6 (5.0)	197	0.9 (6.5)	1.01	0.98–1.04	0.565
Sulpiride	35	2.1 (8.2)	411	1.7 (6.6)	1.00	0.97–1.04	0.858
Clozapine	5	0.2 (1.4)	273	0.9 (4.5)	0.93	0.82–1.05	0.253
Olanzapine	4	0.2 (1.9)	207	1.5 (7.9)	0.91	0.80–1.04	0.182
Quetiapine	17	0.9 (5.1)	181	1.0 (6.5)	1.01	0.98–1.04	0.563
Zotepine	7	0.4 (3.0)	123	0.4 (2.8)	1.03	0.97–1.10	0.349
Risperidone	25	0.8 (3.9)	576	1.8 (6.1)	0.91	0.82–1.01	0.063
Amisulpride	9	0.7 (3.8)	109	0.9 (6.5)	0.99	0.95–1.04	0.674
Aripiprazole	6	0.5 (3.1)	45	0.2 (2.1)	1.07*	1.01–1.13	0.025

*P<0.05.

**P<0.01.

^a^Adjusted for Charlson comorbidity index at the first admission and the following variables within 180 days before the index date, including the number of psychiatric hospital admissions, substance use disorders, physical illnesses, and concomitant medications (listed in Table 1).

### Effect of continuous antipsychotic treatment on acute coronary syndrome

Table 3 shows that, in current users, three individual antipsychotic agents were associated with increased risk in the first 30 days of starting use prior to the index date, including aripiprazole, chlorpromazine, and haloperidol for which the adjusted risk ratios were 3.56 (p < 0.05), 3.04 (p = 0.004), and 2.46 (p < 0.05), respectively. Nevertheless, with longer use of these three drugs, the risk decreased.

**Table 3:** Risk of acute coronary syndrome and effect of cumulative days of continuous antipsychotic treatment (no use as the reference group).

	Cases (n = 147)	Controls (n = 2940)	Multivariate Adjusted Model		
Characteristic, n (%)			Adjusted risk ratio^a^	95% CI	P-value
Chlorpromazine					
No use	93 (63.3)	2064 (70.2)	Reference	–	–
>30 days	39 (26.5)	767 (26.1)	1.11	0.68–1.81	0.671
0–30 days	15 (10.2)	109 (3.7)	3.04**	1.42–6.50	0.004
Haloperidol					
No use	19 (12.9)	524 (17.8)	Reference	–	–
>30 days	88 (59.9)	2012 (68.4)	1.25	0.64–2.44	0.506
0–30 days	40 (27.2)	404 (13.7)	2.46*	1.13–5.36	0.024
Flupentixol					
No use	91 (61.9)	1734 (59.0)	Reference	–	–
>30 days	45 (30.6)	1009 (34.3)	0.68	0.42–1.09	0.106
0–30 days	11 (7.5)	197 (6.7)	1.01	0.41–2.51	0.985
Sulpiride					
No use	32 (21.8)	761 (25.9)	Reference	–	–
>30 days	80 (54.4)	1768 (60.1)	1.01	0.59–1.72	0.980
0–30 days	35 (23.8)	411 (14.0)	1.73	0.89–3.38	0.108
Clozapine					
No use	134 (91.2)	2448 (83.3)	Reference	–	–
>30 days	8 (5.4)	219 (7.5)	0.68	0.28–1.62	0.381
0–30 days	5 (3.4)	273 (9.3)	0.38	0.13–1.09	0.073
Olanzapine					
No use	108 (73.5)	2170 (73.8)	Reference	–	–
>30 days	35 (23.8)	563 (19.2)	1.16	0.70–1.92	0.559
0–30 days	4 (2.7)	207 (7.0)	0.12	0.01–1.89	0.132
Quetiapine					
No use	94 (64.0)	2233 (76.0)	Reference	–	–
>30 days	36 (24.5)	526 (17.9)	1.26	0.76–2.09	0.366
0–30 days	17 (11.6)	181 (6.2)	1.99	0.96–4.10	0.063
Zotepine					
No use	112 (76.2)	2321 (79.0)	Reference	–	–
>30 days	28 (19.1)	496 (16.9)	0.99	0.57–1.72	0.978
0–30 days	7 (4.8)	123 (4.2)	1.29	0.49–3.37	0.606
Risperidone					
No use	59 (40.1)	1172 (39.9)	Reference	–	–
>30 days	63 (42.9)	1192 (40.5)	0.72	0.44–1.17	0.186
0–30 days	25 (17.0)	576 (19.6)	0.58	0.30–1.13	0.111
Amisulpride					
No use	128 (87.1)	2612 (88.8)	Reference	–	–
>30 days	10 (6.8)	219 (7.5)	0.91	0.41–2.02	0.811
0–30 days	9 (6.1)	109 (3.7)	0.91	0.32–2.54	0.851
Aripiprazole					
No use	137 (93.2)	2783 (94.7)	Reference	–	–
>30days	4 (2.7)	112 (3.8)	0.41	0.09–1.79	0.235
0–30 days	6 (4.1)	45 (1.5)	3.56*	1.24–10.27	0.019

*P<0.05.

**P<0.01.

^a^Adjusted for Charlson comorbidity index at the first admission and the following variables within 180 days before the index date, including the number of psychiatric hospital admissions, substance use disorders, physical illnesses, and concomitant medications (listed in Table 1).

### Sensitivity analysis

We conducted sensitivity analyses to validate the risks of acute coronary syndrome. The propensity–score-adjusted model showed no significant difference compared to the associations estimated based on the multivariate regression model (Table A in S1 File). For example, relative to noncurrent users, the adjusted risk ratios of aripiprazole were 3.59 (95% CI = 1.18–10.95, p < 0.05) in the propensity score-adjusted model and 3.68 (95% CI = 1.27–10.64, p < 0.05) in the multivariate-adjusted model.

In the subgroup analyses, current use of chlorpromazine was associated with the risk of developing acute myocardial infarction (ICD 9 codes 410.xx) (adjusted RR = 2.79, p < 0.05); aripiprazole had an adjusted RR of 4.39, p = 0.068 (Table B in S1 File). These results were similar to the risk of acute coronary syndrome associated with all cases.

Furthermore, when we did the sensitivity analyses with a p-value of 0.01 set as significant, the interpretations of the results did not change significantly. For example, in Fig 1, chlorpromazine and haloperidol were still associated with the risk, but aripiprazole was not. Additionally, this study was the first investigation to explore this association in the schizophrenia. Thus, based on the exploratory nature of this research, we used a p-value of 0.05 as significant in the main analyses.

## Discussion

We used the strictest criteria [10, 25] to identify the cases of acute coronary syndrome in a population-based cohort of schizophrenia inpatients to estimate the associations of individual antipsychotics and the risk of acute coronary syndrome. We investigated whether or not a temporal relationship existed for each of the antipsychotic drugs and acute coronary syndrome that could provide support for a causal relationship. Furthermore, we explored dose dependency relationships and the risk of acute coronary syndrome when an antipsychotic drug was taken in the short-term. Aripiprazole, a second-generation antipsychotic, was potentially significantly associated with the risk of acute coronary syndrome. Two first-generation antipsychotics, chlorpromazine and haloperidol, were also associated with significant risk.

### Explanation of the associations between antipsychotics and acute coronary syndrome

The mechanisms remain speculative. The affinities for neurotransmitter receptors (Table C in S1 File) could help explain the mechanism. Aripiprazole, identified as having a potentially elevated risk, is partially agonistic for dopamine D2 receptors and is antagonistic of serotonergic 5-HT2 receptors [30]. However, aripiprazole acts at the D2 receptors and does not affect 5-HT receptors at therapeutic doses [30]. Therefore, the mechanism of the strong D2 receptor and weak 5-HT receptor affinities could explain why the risk exists. It is compatible with the two other antipsychotics (i.e., chlorpromazine, haloperidol) identified with the risk also have strong dopamine D2 and weak serotonergic 5-HT receptor affinities. We propose that the identified drugs that carry a risk of acute coronary syndrome primarily block dopamine receptors, leading to an imbalance between the dopamine and serotonin systems, predisposing patients to platelet aggregation.

### Association between metabolic syndrome and acute coronary syndrome

Several proxies for metabolic syndrome were identified with the risk of acute coronary syndrome in the study, including diabetes mellitus, hypertension, and hyperlipidemia (more case patients used lipid modifying agents than control patients) (Table 1). The findings are consistent with a prior study [12] reporting that in patients with schizophrenia, metabolic syndrome is associated with coronary heart disease. Several studies [31, 32] have shown that certain second-generation antipsychotics, especially clozapine and olanzapine, are associated with increased rates of metabolic syndrome, which could contribute to the risk of acute coronary syndrome. However, the present study showed that second-generation antipsychotics had an overall protective effect on acute coronary syndrome. Among the second-generation antipsychotics, aripiprazole showed a significant risk effect on acute coronary syndrome, whereas clozapine and olanzapine had protective effects but without statistical significance. Thus, even clozapine and olanzapine could induce metabolic syndrome; however, in this study, they were not associated with elevated risk of acute coronary syndrome. The mechanisms deserve future study. Due to the small sample size in some of the exposure groups, future investigations are also needed to confirm the results.

### Limitations

This study is limited in several ways. First, several potential confounding factors could have affected the risks of acute coronary syndrome, such as lifestyles, substance use problems, body mass index, and smoking, which were not available in the database. Residual confounding due to unmeasured covariates is, therefore, possible. Second, we were unable to assess adherence to the prescribed medication because drug use data were obtained from claims databases. However, non-adherence to the prescribed therapeutic regimen would most likely result in underestimation of the actual risk based on non-differentiated misclassification of exposure [33]. Third, we enrolled the cases with incident acute coronary syndrome requiring hospitalization and excluded those with milder forms of coronary heart disease. This might have caused underestimation of the incidence of occurrence. Fourth, the number of cases in this study was modest. Among the cases, the number of patients using an individual antipsychotic could be small (such as for aripiprazole, which appears to show some signal, but is based only on 6 exposed cases) and the estimated confidence interval of risk could be wide. Therefore, we analyzed dose-dependency relationships to estimate these associations. However, the inferences of the findings would be better conservative. Further studies are needed to validate the findings because of the exploratory nature of the study. Fifth, few of patients could use more than one antipsychotic agent in this study. We did not analyze the risk of multiple antipsychotics use due to the small number in the particular combined set of antipsychotics (such as haloperidol combined with sulpiride).

## Conclusions

Current use of aripiprazole, chlorpromazine, and haloperidol could be potentially associated with the risk of acute coronary syndrome, particularly at the start of therapy.

## Supporting Information

S1 File **Fig A.** Study flow diagram. **Table A.** The distribution of current use of second-generation and first-generation antipsychotics between the case patients with acute coronary syndrome and controls among patients with schizophrenia (noncurrent use as the reference). **Table B.** The distribution of second- and first-generation antipsychotic drug use between the case patients with acute myocardial infarction (ICD 9 codes 410.xx) and controls among the cohort with schizophrenia (N = 31,177). **Table C.** Relative neurotransmitter receptor affinities for antipsychotics at therapeutic doses, information adapted from the references and modified.
(DOC)
