# Supplementary material for: Antipsychotic Medications and Risk of Acute Coronary Syndrome in Schizophrenia: A Nested Case-Control Study
Source: PLoS One. 2016 Sep 22;11(9):e0163533. doi: 10.1371/journal.pone.0163533 (PMC5033466; doi:10.1371/journal.pone.0163533)
Supplement: S1 File — Fig A. Study flow diagram. Table A. The distribution of current use of second-generation and first-generation antipsychotics between the case patients with acute coronary syndrome and controls among patients with schizophrenia (noncurrent use as the reference). Table B. The distribution of second- and first-generation antipsychotic drug use between the case patients with acute myocardial infarction (ICD 9 codes 410.xx) and controls among the cohort with schizophrenia (N = 31,177). Table C. Relative neurotransmitter receptor affinities for antipsychotics at therapeutic doses, information adapted from the references and modified. (DOC) [file pone.0163533.s001.doc]

S1 Fig A. Study flow diagram

Patients with the consistent diagnosis of schizophrenia (ICD-9 code: 295.**) (N=38,120)

Patients aged between 15 and 65 years, final study cohort (N=33,024)

Patients with incident hospitalized acute coronary syndrome (n=148) (ICD 9: 410.xx, 411.1x) after their first admissions.

Broad search through all claims data between 2000 and 2010

Nationwide Psychiatric Inpatient Medical Claims (PMID) in Taiwan (ICD-9 code of 290.**~319.**) (1996-2008) (N=187,117)

Patients first discharged with mental disorder diagnosis (ICD-9 code of 290.**~319.**), from 2000 to 2008 (N=125,225) (no any psychiatric admission between 1996 and 1999)

Each case was matched with 20 controls. 147 valid case-control due to unavailability of controls for one case

The study cohort (N=31,177), excluding the diagnoses of ischemic heart diseases, myocardial infarction, coronary artery diseases (ICD9: 410-414) before the first admission

**S1 Table A. The distribution of current use of second-generation and first-generation antipsychotics between the case patients with acute coronary syndrome and controls among patients with schizophrenia (noncurrent use as the reference)**

|  |  | Cases  (N = 147) | Controls  (N = 2940) |  | Propensity | score-adjusted model |  | Multivariate | adjusted model |
| --- | --- | --- | --- | --- | --- | --- | --- | --- | --- |
| Characteristic |  | N (%) | N (%) |  | Risk Ratio^a^ | 95% CI |  | Risk Ratio^b^ | 95% CI |
| Chlorpromazine |  |  |  |  |  |  |  |  |  |
| Current use |  | 15 (10.2) | 109 (3.7) |  | 3.07** | 1.45-6.53 |  | 2.96** | 1.40-6.24 |
| Haloperidol |  |  |  |  |  |  |  |  |  |
| Current use |  | 40 (27.2) | 404 (13.7) |  | 2.08** | 1.22-3.56 |  | 2.03** | 1.20-3.44 |
| Flupentixol |  |  |  |  |  |  |  |  |  |
| Current use |  | 11 (7.5) | 197 (6.7) |  | 1.29 | 0.54-3.13 |  | 1.18 | 0.49-2.87 |
| Sulpiride |  |  |  |  |  |  |  |  |  |
| Current use |  | 35 (23.8) | 411 (14.0) |  | 1.68 | 0.98-2.87 |  | 1.72* | 1.01-2.94 |
|  |  |  |  |  |  |  |  |  |  |
| Clozapine |  |  |  |  |  |  |  |  |  |
| Current use |  | 5 (3.4) | 273 (9.3) |  | 0.39 | 0.14-1.12 |  | 0.40 | 0.14-1.13 |
| Olanzapine |  |  |  |  |  |  |  |  |  |
| Current use |  | 4 (2.7) | 207 (7.0) |  | 0.00 | 0.00- |  | 0.00 | 0.00- |
| Quetiapine |  |  |  |  |  |  |  |  |  |
| Current use |  | 17 (11.6) | 181 (6.2) |  | 1.84 | 0.91-3.71 |  | 1.85 | 0.91-3.74 |
| Zotepine |  |  |  |  |  |  |  |  |  |
| Current use |  | 7 (4.8) | 123 (4.2) |  | 1.28 | 0.49-3.31 |  | 1.29 | 0.50-3.36 |
| Risperidone |  |  |  |  |  |  |  |  |  |
| Current use |  | 25 (17.0) | 576 (19.6) |  | 0.72 | 0.39-1.32 |  | 0.71 | 0.38-1.29 |
| Amisulpride |  |  |  |  |  |  |  |  |  |
| Current use |  | 9 (6.1) | 109 (3.7) |  | 1.02 | 0.37-2.79 |  | 0.91 | 0.33-2.56 |
| Aripiprazole |  |  |  |  |  |  |  |  |  |
| Current use |  | 6 (4.1) | 45 (1.5) |  | 3.59* | 1.18-10.95 |  | 3.68* | 1.27-10.64 |
|  |  |  |  |  |  |  |  |  |  |

**P* < .05, ***P* < .01.

^a^Adjusted with the propensity score, described in the Methods.

^b^Adjusted for Charlson comorbidity index at the first admission, and the following variables within 180 days before the index date, including the number of psychiatric hospital admissions, physical illnesses and concomitant medications (listed in Table 1), and the numbers of mood stabilizer, second-, and first-generation antipsychotics respectively.

S1 Table B. The distribution of second- and first-generation antipsychotic drug use between the case patients with acute myocardial infarction (ICD 9 codes 410.xx) and controls among the cohort with schizophrenia (N=31,177)

|  |  | Cases  (n=100) | Controls (n=2000) |  |  | Multivariate  Model | adjusted |
| --- | --- | --- | --- | --- | --- | --- | --- |
| Characteristic, N (%) |  |  |  |  | Adjusted Risk Ratio^a^ | 95% CI | *P* |
| Any use of first-generation antipsychotics |  |  |  |  |  |  |  |
| Non-current use |  | 44 (44.0) | 1305 (65.3) |  | Reference | - | - |
| Current use |  | 56 (56.0) | 695 (34.8) |  | 2.04** | 1.19-3.51 | 0.010 |
| Chlorpromazine |  |  |  |  |  |  |  |
| Non-current use |  | 90 (90.0) | 1926 (96.3) |  | Reference | - | - |
| Current use |  | 10 (10.0) | 74 (3.7) |  | 2.79* | 1.04-7.51 | 0.042 |
| Haloperidol |  |  |  |  |  |  |  |
| Non-current use |  | 75 (75.0) | 1733 (86.7) |  | Reference | - | - |
| Current use |  | 25 (25.0) | 267 (13.4) |  | 1.70 | 0.86-3.35 | 0.129 |
| Flupentixol |  |  |  |  |  |  |  |
| Non-current use |  | 93 (93.0) | 1852 (92.6) |  | Reference | - | - |
| Current use |  | 7 (7.0) | 148 (7.4) |  | 1.07 | 0.37-3.09 | 0.895 |
| Sulpiride |  |  |  |  |  |  |  |
| Non-current use |  | 77 (77.0) | 1723 (86.2) |  | Reference | - | - |
| Current use |  | 23 (23.0) | 277 (13.9) |  | 1.46 | 0.73-2.91 | 0.283 |
|  |  |  |  |  |  |  |  |
| Any use of second-generation antipsychotics |  |  |  |  |  |  |  |
| Non-current use |  | 63 (63.0) | 1042 (52.1) |  | Reference | - | - |
| Current use |  | 37 (37.0) | 958 (47.9) |  | 0.49* | 0.27-0.87 | 0.016 |
| Clozapine |  |  |  |  |  |  |  |
| Non-current use |  | 98 (98.0) | 1818 (90.9) |  | Reference | - | - |
| Current use |  | 2 (2.0) | 182 (9.1) |  | 0.38 | 0.09-1.62 | 0.191 |
| Olanzapine |  |  |  |  |  |  |  |
| Non-current use |  | 96 (96.0) | 1861 (93.1) |  | Reference | - | - |
| Current use |  | 4 (4.0) | 139 (7.0) |  | 0.00 | 0.00- | 0.988 |
| Quetiapine |  |  |  |  |  |  |  |
| Non-current use |  | 94 (94.0) | 1887 (94.4) |  | Reference | - | - |
| Current use |  | 6 (6.0) | 113 (5.7) |  | 0.54 | 0.12-2.42 | 0.420 |
| Zotepine |  |  |  |  |  |  |  |
| Non-current use |  | 96 (96.0) | 1912 (95.6) |  | Reference | - | - |
| Current use |  | 4 (4.0) | 88 (4.4) |  | 1.05 | 0.30-3.72 | 0.938 |
| Risperidone |  |  |  |  |  |  |  |
| Non-current use |  | 87 (87.0) | 1602 (80.1) |  | Reference | - | - |
| Current use |  | 13 (13.0) | 398 (19.9) |  | 0.57 | 0.25-1.30 | 0.185 |
| Amisulpride |  |  |  |  |  |  |  |
| Non-current use |  | 93 (93.0) | 1930 (96.5) |  | Reference | - | - |
| Current use |  | 7 (7.0) | 70 (3.5) |  | 1.00 | 0.29-3.37 | 0.996 |
| Aripiprazole |  |  |  |  |  |  |  |
| Non-current use |  | 97 (97.0) | 1978 (98.9) |  | Reference | - | - |
| Current use |  | 3 (3.0) | 22 (1.1) |  | 4.39 | 0.90-21.43 | 0.068 |

^a^Adjusted for Charlson comorbidity index at the first admission, and the following variables within 180 days before the index date, including the number of psychiatric hospital admissions, substance use disorders, physical illnesses, and concomitant medications (listed in Table 1) respectively.

S1 Table C. Relative neurotransmitter receptor affinities for antipsychotics at therapeutic doses, information adapted from the references[^1-6^](#_ENREF_1) and modified

| Receptors |  | Clozapine |  | Olanzapine |  | Quetiapine |  | Zotepine |  | Risperidone |  | Amisulpride |  | Aripiprazole |  | Chlorpromazine |  | Haloperidol |  | Flupentixol |  | Sulpride |
| --- | --- | --- | --- | --- | --- | --- | --- | --- | --- | --- | --- | --- | --- | --- | --- | --- | --- | --- | --- | --- | --- | --- |
| D_1_ |  | + |  | + + |  | – |  | + |  | + |  | – |  | – |  | + |  | + |  | + |  | + |
| D_2_ |  | + |  | + + |  | + |  | + + |  | + + + |  | + + + + |  | + + + + |  | + + + |  | + + + + |  | + + + + |  | + + |
| D_3_ |  | + |  | + |  | – |  | + + |  | + + |  | + + |  | + + |  |  |  | + + + |  |  |  |  |
| D_4_ |  | + + |  | + + |  | – |  | + |  | – |  | – |  | + |  | + + |  | + + + |  | + + |  | – |
| 5-HT_1A_ |  | – |  | – |  | – |  | + + |  | – |  |  |  | + +^a^ |  | – |  | – |  | – |  | – |
| 5-HT_2A_ |  | + + + |  | + + + |  | + + |  | + + + |  | + + + + |  | – |  | + + + ^a^ |  | + |  | + |  | + + |  | – |
| 5-HT_2C_ |  | + + |  | + + |  | – |  | + + |  | + + |  | – |  | + ^a^ |  |  |  | – |  |  |  | – |
| 5-HT_6_ |  | + + |  | + + |  | – |  | + + |  | － |  |  |  | + ^a^ |  |  |  | – |  | ++ |  | – |
| 5-HT_7_ |  | + + |  | – |  | – |  | + + |  | + + + |  |  |  | + + ^a^ |  | – |  |  |  |  |  |  |
| α_1_ |  | + + + |  | + + |  | + + + |  | + + |  | + + + |  | – |  | + |  | + + + |  | + + + |  | + + + |  | – |
| α_2_ |  | + |  | + |  | – |  | + + |  | + + |  | – |  | + |  | – |  | – |  | – |  | – |
| H_1_ |  | + + + |  | + + + |  | + + + |  | + + |  | – |  | – |  | + |  | + + + |  | – |  | + + |  | – |
| M_1_ |  | + + + + |  | + + + |  | + + |  | + |  | – |  | – |  | – |  | + + |  | – |  | – |  | – |
|  |  |  |  |  |  |  |  |  |  |  |  |  |  |  |  |  |  |  |  |  |  |  |

Affinity: –: minimal to none; +: low; + +: moderate; + + +: high; + + + +: very high; cells are empty when no data were available.

H1: histamine-1 receptor subtype; M1: muscarinic-1 receptor subtype.

D1: dopamine-1 receptor subtype; D2: dopamine-2 receptor subtype; D3: dopamine-3 receptor subtype; D4: dopamine-4 receptor subtype;

5-HT1: 5-hydroxytryptamine-1 subtype; 5-HT2: 5-hydroxytryptamine-2 subtype; 5-HT3: 5-hydroxytryptamine-3 subtype; 5-HT4: 5-hydroxytryptamine-4 subtype; 5-HT5: 5-hydroxytryptamine-5 subtype; 5-HT6: 5-hydroxytryptamine-6 subtype; 5-HT7: 5-hydroxytryptamine-7 subtype

α1: adrenergic-1 receptor subtype; α2: adrenergic-2 receptor subtype;

^a^The review report [^7^](#_ENREF_7) suggests that aripiprazole acts as a selective partial agonist at the dopamine D(2) receptor and does not affect 5-HT receptors at therapeutic doses.

**References**

1. Abi-Dargham A, Laruelle M. Mechanisms of action of second generation antipsychotic drugs in schizophrenia: insights from brain imaging studies. Eur Psychiatry 2005;20(1):15-27.

2. Miyamoto S, Duncan GE, Marx CE, Lieberman JA. Treatments for schizophrenia: a critical review of pharmacology and mechanisms of action of antipsychotic drugs. Mol Psychiatry 2005;10(1):79-104.

3. Arnt J, Skarsfeldt T. Do novel antipsychotics have similar pharmacological characteristics? A review of the evidence. Neuropsychopharmacology 1998;18(2):63-101.

4. Bymaster FP, Calligaro DO, Falcone JF, et al. Radioreceptor binding profile of the atypical antipsychotic olanzapine. Neuropsychopharmacology 1996;14(2):87-96.

5. Wu CS, Wang SC, Gau SS, Tsai HJ, Cheng YC. Association of stroke with the receptor-binding profiles of antipsychotics-a case-crossover study. Biological psychiatry 2013;73(5):414-421.

6. Meltzer HY, Matsubara S, Lee JC. Classification of typical and atypical antipsychotic drugs on the basis of dopamine D-1, D-2 and serotonin2 pKi values. The Journal of pharmacology and experimental therapeutics 1989;251(1):238-246.

7. Wood M, Reavill C. Aripiprazole acts as a selective dopamine D2 receptor partial agonist. Expert Opin Investig Drugs 2007;16(6):771-775.
